# A Polymer‐Oriented Self‐Assembly Strategy toward Mesoporous Metal Oxides with Ultrahigh Surface Areas

**DOI:** 10.1002/advs.201801543

**Published:** 2019-01-28

**Authors:** Hailong Xiong, Tunan Gao, Kaiqian Li, Yali Liu, Yali Ma, Jingwei Liu, Zhen‐An Qiao, Shuyan Song, Sheng Dai

**Affiliations:** ^1^ State Key Laboratory of Inorganic Synthesis and Preparative Chemistry Jilin University Changchun Jilin 130012 China; ^2^ Key Laboratory of Rare Earth Chemistry and Physics Changchun Institute of Applied Chemistry Graduate School of the Chinese Academy of Sciences Chinese Academy of Sciences Changchun Jilin 130022 China; ^3^ Chemical Sciences Division Oak Ridge National Laboratory Oak Ridge TN 37831 USA

**Keywords:** high crystallinity, mesoporous metal oxides, photocatalytic hydrogen production, polymer‐oriented self‐assembly strategy, ultrahigh surface area

## Abstract

Mesoporous metal oxides (MMOs) have attracted comprehensive attention in many fields, including energy storage, catalysis, and separation. Current synthesis of MMOs mainly involve use of surfactants as templates to generate mesopores and organic reagents as solvents to hinder hydrolysis and condensation of inorganic precursors, which is adverse to adjusting the interactions between surfactants and inorganic precursors. The resulting products have uncontrollable pore structure, crystallinity, and relatively lower surface areas. Here, a facile and general polymer‐oriented self‐assembly strategy to synthesize a series of MMOs (e.g., TiO_2_, ZrO_2_, NbO_5_, Al_2_O_3_, Ta_2_O_5_, HfO_2_, and SnO_2_) by using cationic polymers as porogens and metal alkoxides as metal oxide precursors in a robust aqueous synthesis system are reported. Nitrogen adsorption analysis and transmission electron microscopy confirm that the obtained MMOs have ultrahigh specific surface areas and large pore volumes (i.e., 733 m^2^ g^−1^ and 0.485 cm^3^ g^−1^ for mesoporous TiO_2_). Moreover, the structural parameters (surface area, pore size, and pore volume) and crystallinity can be readily controlled by tuning the interactions between cationic polymers and precursors. The as‐synthesized crystalline mesoporous TiO_2_ exhibits promising performance in photocatalytic water splitting of hydrogen production and a high hydrogen production rate of 3.68 mol h^−1^ g^−1^.

## 1 Introduction

Mesoporous metal oxides (MMOs) have been of great interest over the past decades for comprehensive applications including energy storage, selective oxidation, adsorption, and gas sensor.^1^, ^2^, ^3^, ^4^, ^5^, ^6^, ^7^, ^8^, ^9^, ^10^, ^11^ Most of these applications are strongly relied on their superior physical, chemical properties, and the combination of their advanced structural properties such as high specific surface areas, large pore volumes, and narrow pore size distributions.^12^ Various synthetic methods have been developed to synthesize MMOs. One promising route is nanocasting method,^13^, ^14^, ^15^, ^16^, ^17^, ^18^, ^19^, ^20^, ^21^ which depends on the usage of mesoporous silica, mesoporous carbon, or polymer as hard templates to provide the desired mesoporous structure and subsequent removal of templates by etching or calcination to finally obtain MMOs. However, the application of this method suffers from some limitations, for instance, the mesoporous characteristics of such oxides are completely relied on the framework of hard templates. Moreover, there are only a few templates available for use, and the procedure is tedious, costly as well as not suitable for mass production, which greatly hinders their practical applications.

To overcome these obstacles, a molecular self‐assembly method based on cooperative organization of surfactant templates and inorganic precursors has been put forward, called evaporation‐induced self‐assembly (EISA) method.^22^, ^23^, ^24^, ^25^, ^26^, ^27^, ^28^, ^29^, ^30^, ^31^, ^32^ The key issue of EISA method is a controllable and slow hydrolysis–condensation process of metal oxide precursors catalyzed by acid or base in an organic solvent synthesis system.^33^ The interactions between surfactants and inorganic precursors are also critical for the formation of mesostructures, including electrostatic interactions (S^+^ I^−^, S^−^ I^+^, S^+^ X^−^ I^+^, S^−^ X^+^ I^−^), where S represents the surfactant templates, I is the inorganic species, and X is a mediator, ligand–metal interactions, coordination type interactions, and so on.^34^ In the past of years, Zhao's group employed traditional triblock copolymer (e.g., Pluronic F127, P123) and diblock copolymer poly (ethylene oxide)‐*b*‐polystyrenem (PEO‐*b*‐PS) as the structure‐directing agents to successfully prepare a series of highly ordered MMOs (e.g., TiO_2_, N_2_O_5_, WO_3_, In_2_O_3_, and Al_2_O_3_) by the EISA method.^35^, ^36^ By using acetate groups as a complexing agent to control the hydrolysis and condensation of metal alkoxides, Fan et al. synthesized multicomponent MMOs in a sol–gel solution composed of hydrochloric acid, acetic acid, and ethanol.^37^ Suib and co‐workers reported a sol–gel‐based inverse micelle method through hydrogen bonding, which gave direct access to crystalline and thermally stable mesoporous materials by using HNO_3_ to prevent the condensation of metal precursors and balance the charge of reaction system.^38^ Despite the great success of EISA approach, it also has its deficiencies. For example, the synthesis based on complicated sol–gel processes is quite sensitive to experimental conditions, such as temperature, pH, relative humidity, solvent purity, and so forth, which can significantly affect the hydrolysis–condensation process of metal oxide precursors. Introducing plenty of organic solvents plus an acid or an additive agent to hinder hydrolysis and condensation results in the synthesis process laborious, cost‐consuming and time‐consuming.^39^, ^40^, ^41^ Moreover, it is really of hardship to adjust the interactions between surfactants and inorganic precursors in organic solvent synthesis system, which leads to the crystallinity, morphology, and porous structure of products uncontrollable.^39^ Above all, the resulting MMOs by EISA method have relatively lower surface areas, usually lower than 250 m^2^ g^−1^.

**Scheme 1:**
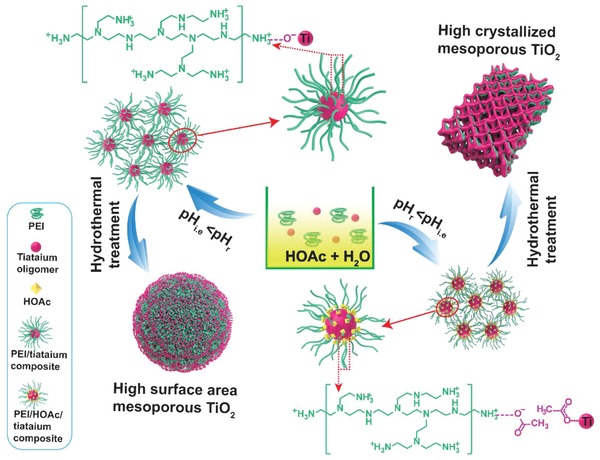
Schematic representation of tentative formation mechanism of mesoporous TiO_2_ through the polymer‐oriented self‐assembly strategy.

Here, we demonstrate a facile, versatile, yet unexplored method, referred to as a polymer‐oriented self‐assembly strategy, for the preparation of a series of MMOs (e.g., TiO_2_, ZrO_2_, NbO_5_, Al_2_O_3_, Ta_2_O_5_, HfO_2_, and SnO_2_) with ultrahigh surface areas and monomodal pore sizes by using metal alkoxides as metal oxide precursors in a robust aqueous synthesis system (**Scheme** **1**). In this approach, commercial cationic polymers, polyethylenimine (PEI), or polydimethyldiallylammonium chloride (PDADMAc) have been employed as porogens on the mesoscale to produce abundant mesopores. Acetic acid (HOAc) not only acts as a pH regulator to dictate the type of interactions between titanium species and polymers, but also as a coordination agent to modify condensation kinetics of metal alkoxides and balance charge of reaction system. The structural properties (e.g., porous architectures, crystal phases) and morphologies of obtained MMOs can be easily adjusted by tuning the pH of reaction system (pH_r_). When pH_r_ > pH_i.e_ (isoelectric point of metal oxides), the positively charged PEI (S^+^) interacts with anionic metal oligomers through an electrostatic interaction (S^+^ I^−^) and thus triggers the organic–inorganic assembly into ultrahigh surface area MMOs. The resulting MMOs have ultrahigh surface areas, 733 m^2^ g^−1^ for TiO_2_, 422 m^2^ g^−1^ for ZrO_2_, 360 m^2^ g^−1^ for Nb_2_O_5_, 393 m^2^ g^−1^ for Al_2_O_3_, 190 m^2^ g^−1^ for Ta_2_O_5_, 344 m^2^ g^−1^ for HfO_2_, and 174 m^2^ g^−1^ for SnO_2_. When pH_r_ < pH_i.e_, highly crystallized mesoporous materials can be prepared in the presence of a type of S^+^ X^−^ I^+^ organic–inorganic interaction mode, where X represents acetate ions (CH_3_COO^−^). The as‐synthesized crystalline mesoporous TiO_2_ with a relatively high surface area shows properties that are promising application in photocatalytic water splitting of hydrogen production, a high photocatalytic hydrogen generation rate of 3.68 mol h^−1^ g^−1^, and exhibits great potential for anticipative applications in gas sensor, energy storage, and catalysis.

## 2 Results and Discussion

A series of MMOs with ultrahigh surface areas have been synthesized by the polymer‐oriented self‐assembly strategy in a robust aqueous synthesis system. Mesoporous TiO_2_ labeled as MT*x*, *x* referred to the amount of HOAc, was took as a representative in the following discussion. In this approach, tetrabutyl titanate (TBOT) first hydrolyzed in H_2_O/PEI/HOAc solution under stirring at room temperature for 2 h to form mesoporous titanium/PEI composites (Figure S1 and Table S1, Supporting Information). Then, the resulting titanium/PEI composites were then hydrothermally treated at 100 °C for cross‐linking and condensation of titanium oligomers. Finally, the obtained composites were washed with distilled water and absolute ethanol several times in sequence to remove PEI, giving rise to mesoporous TiO_2_. When the amount of HOAc ranged from 0 to 1.5 mL, the ultrahigh surface area mesoporous TiO_2_ could be obtained. When the amount of HOAc was larger than 2.5 mL, we could get mesoporous TiO_2_ with highly crystallized framework. It was noteworthy that the yield of products was as high as ≈95% and grams of samples could be easily synthesized in one bath.

**Figure 1:**
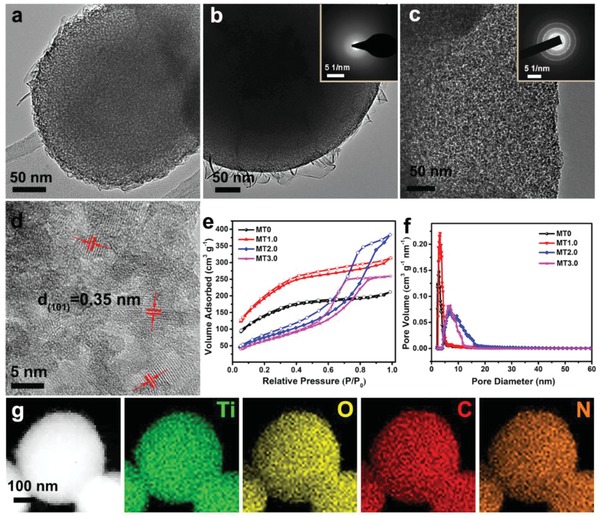
a–c) TEM images and d) HRTEM image of mesoporous TiO_2_: MT0 (a), MT1.0 (b), and MT3.0 (c,d). The insets in (b) and (c) are SAED patterns of MT1.0 and MT3.0. e) Nitrogen sorption isotherms and f) pore size distributions of mesoporous TiO_2_: MT0, MT1.0, MT2.0, and MT3.0. g) Scanning TEM image and the corresponding EDS mapping images of elemental Ti, O, C, and N for MT1.0.

The effect of HOAc content on the mesostructure and morphology of mesoporous TiO_2_ was first investigated by transmission electron microscopy (TEM) and scanning electron microscopy (SEM). When HOAc content was less than 1.5 mL, MT0 and MT1.0 were used as representatives, SEM images (Figure S2a,b, Supporting Information) of MT0 and MT1.0 disclosed approximate microscale spheres with partial connections. TEM images (**Figure** **1**a,b) of these two samples exhibited a large number of uniform mesopores with an open pore structure and the pore sizes were estimated to be about 2.6–3.2 nm. High‐resolution TEM (HRTEM) image (Figure S2c, Supporting Information) and selected‐area electron diffraction (SAED) pattern (inset of Figure 1b) of MT1.0 confirmed the amorphous nature. Besides, there were a few nanosheets coating outside the TiO_2_ microsphere, which was attributed to further growth and condensation of titanium oligomers during hydrothermal process. To study the element distribution and chemical composition for MT1.0, EDX spectra (Figure S2d, Supporting Information) combined with scanning TEM and EDS mapping images (Figure 1g) were measured, showing the uniform distribution of Ti, O, C, and N. The presence of carbon and nitrogen phases indicated it still had residual PEI molecules in mesoporous TiO_2_ after washing with water and absolute ethanol, which was beneficial for maintaining the stability of mesostructure. When HOAc content was more than 2.5 mL, the pore structure and morphology were significantly different from MT1.0. MT2.5 and MT3.0 showed similar irregular bulk morphologies with rough surfaces in SEM images (Figure S3a,b, Supporting Information), implying the existence of abundant pores. According to TEM images (Figure S3c, Supporting Information; Figure 1c), MT2.5 and MT3.0 were both consisted of small TiO_2_ nanocrystals about 8.0 nm and the mesopores about 7.0 nm were interparticle voids. Lattice fringes with a distance of 0.35 nm can be found in HRTEM images (Figure S3d, Supporting Information; Figure 1d), corresponded to d‐spacing of the (101) crystal plane of anatase, which verified the crystalline pore walls of MT2.5 and MT3.0. The continuous sharp circled on the SAED (inset of Figure 1c) further confirmed that MT3.0 had a polycrystalline anatase phase with high crystallinity. When HOAc content was in the range of 1.5 to 2.5 mL, it can be observed that MT2.0 was a mixture consisted of amorphous and crystalline phase (Figure S4a,b, Supporting Information), indicating it was an intermediate phase.

The pore structure of mesoporous TiO_2_ was investigated by nitrogen adsorption–desorption analysis. Figure 1e and Figure S5a (Supporting Information) showed that the nitrogen sorption isotherms of all of samples had typical type IV curves, indicating the existence of mesoporous structure. The sorption isotherms of the products with HOAc content below 1.5 mL exhibited an distinct capillary condensation step at a relative pressure of 0.2–0.4, corresponding to a small pore size of 2.6–3.2 nm (Figure 1f). The specific Brunauer–Emmett–Teller (BET) surface areas (*S*_BET_) and pore volumes of theses samples were as high as 461–733 m^2^ g^−1^ and 0.296–0.629 cm^3^ g^−1^, respectively (Figure S5b, Supporting Information, **Table** **1**, and Figure S2), which were higher than those reported for similar materials (Table S3, Supporting Information). For the samples synthesized with HOAc content above 2.5 mL, the isotherms of these samples exhibited a H2 hysteresis loop along with another typical pore condensation step at a higher relative pressure (*P*/*P*_0_ = 0.4–0.8), reflecting the presence of large mesopores (6.8–7.2 nm). The *S*_BET_ and pore volume of the products were calculated to be in the range of 226–254 m^2^ g^−1^ and 0.328–0.386 cm^3^ g^−1^. The small‐angle X‐ray powder diffraction (XRD) patterns (Figure S6, Supporting Information) of these samples displayed broad diffraction peaks, indicating a homogeneous wormhole‐like mesopores, which were consistent with those observed in TEM images.

**Table 1:** Structural properties of mesoporous metal oxides

Sample	Polymer	*S*_BET_ a) [m^2^ g^−1^]	Pore size [nm]	*V*_T_ b) [cm^3^ g^−1^]
MT0	PEI	461	2.6	0.296
MT1.0	PEI	733	3.2	0.485
MT2.0	PEI	289	7.0	0.577
MT3.0	PEI	226	7.0	0.384
ZrO_2_	PEI	422	3.1	0.593
HfO_2_	PEI	344	2.6	0.195
Nb_2_O_5_	PEI	360	2.6	0.457
Ta_2_O_5_	PEI	190	2.1	0.176
AlOOH	PEI	360	7.0	0.883
Al_2_O_3_	PEI	393	7.0	1.044
SnO_2_	PEI	174	2.6	0.151
TiO_2_	PDADMAc	575	2.6	0.335
ZrO_2_	PDADMAc	328	2.6	0.242

^a)^ BET surface area

^b)^ Total pore volume.

**Figure 2:**
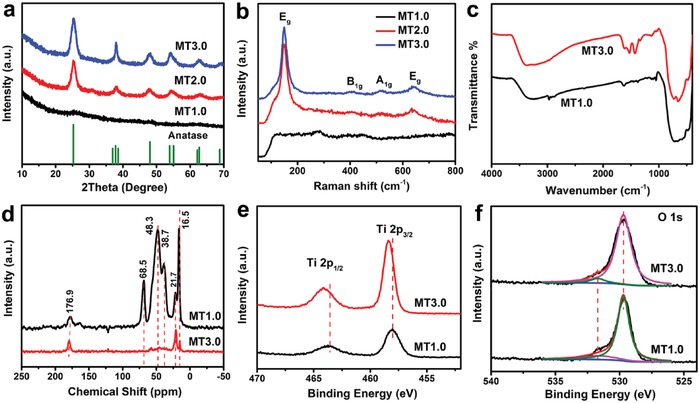
a) XRD patterns and b) Raman spectra of mesoporous TiO_2_: MT1.0, MT2.0, and MT3.0. c) FT‐IR spectra, d) solid state ^13^C NMR spectra, e) Ti 2p, and f) O 1s high‐resolution XPS spectra of mesoporous TiO_2_: MT1.0 and MT3.0.

Wide‐angle XRD analysis was employed to confirm the crystal phase and composition of mesoporous TiO_2_. **Figure** **2**a displayed the transition process from amorphous to crystalline phase with the increase of HOAc content. XRD pattern of MT1.0 showed no obvious diffraction patterns, suggesting its amorphous nature. For MT3.0, all diffraction peaks can be assigned to highly crystalline anatase phase (space group *I41/amd*). The crystal sizes of nanocrystals calculated using the Scherrer equation were about 8.0 nm, which matched well with the observed results from TEM image. Upon further increasing HOAc content, the crystallinity of these products gradually increased (Figure S7, Supporting Information),^42^ which further indicated that HOAc indeed played a crucial part in the crystallinity of mesoporous TiO_2_. Raman test was used to further investigate the crystal phase of mesoporous TiO_2_. Raman spectra (Figure 2b) of MT1.0 showed some weak peaks ranging from 50 to 800 cm^−1^, indicating that its framework was amorphous. While MT2.0 and MT3.0 displayed four anatase Raman transitions, which could be assigned to E_g_ (148.8 cm^−1^), B_1g_ (404.3 cm^−1^), A_1g_ (516.3 cm^−1^), and E_g_ (648.7 cm^−1^), respectively.^43^ The reflected crystallographic microstructures were consistent with HRTEM images and XRD patterns. Above results illustrated that the structural properties (e.g., porous architectures and crystal phases) and morphologies of as‐synthesized samples can be easily controlled by tuning HOAc content.

The structural properties of mesoporous TiO_2_ were elucidated by various spectroscopic measurements. Fourier transform infrared (FT‐IR) spectra, solid‐state ^13^C and ^1^H nuclear magnetic resonance (NMR) spectroscopy were employed to explore the role of HOAc during the synthesis and confirm the presence of PEI in mesoporous TiO_2_. For MT1.0, the characteristic absorptions of FT‐IR spectra (Figure 2c) at 3241 and 1620 cm^−1^ could be assigned to the stretching vibrations of NH groups. The peaks at 1047 cm^−1^ could be attributed to the C–N stretching vibrations.^44^, ^45^ The solid‐state ^13^C NMR spectroscopy (Figure 2d) showed the resonance peaks at 38.7, 41.3, and 68.5 ppm, which matched the methylene groups adjacent to primary, secondary, and tertiary amine groups, respectively. The resonance peak at ≈176.9 ppm was assigned to carbamate, because of CO_2_ adsorption from ambient air by amine groups.^46^ The ^1^H NMR spectroscopy (Figure S8, Supporting Information) showed the peak at 0.5 ppm could be assigned to the methylene groups. For MT3.0, the asymmetric (*v*_as_ = 1520 cm^−1^) and symmetric (*v*_sym_ = 1427 cm^−1^) carboxylate stretches were observed in FT‐IR spectra. The difference between carboxylate stretching frequencies (Δ = *v*_as_ − *v_sym_*) was 93 cm^−1^, suggesting acetate groups bound to metal ions in a bidentate bridging.^47^, ^48^ Moreover, the C–H vibration at 2971 cm^−1^ was almost disappeared. Solid‐state ^13^C NMR spectroscopy showed no resonance peaks in the range of 38–70 ppm. From thermogravimetric analysis (Figure S9a,b, Supporting Information), the total weight loss of MT1.0 and MT3.0 in the range of 150–475 °C was 25.5% and 7.7%, attributed to the decomposition of PEI.^49^ Compared with MT3.0, there were more PEI molecules residues in the framework of MT1.0, which contributed to keeping the stability of mesostructure.

The overall composition of mesoporous TiO_2_ was characterized by X‐ray photoelectron spectroscopy (XPS). XPS survey spectra (Figure S10a, Supporting Information) showed four similar peaks due to species containing Ti, O, C, and N elements in all samples. The Ti 2p spectra (Figure 2e) for MT3.0 presented two peaks at binding energy of 458.5 and 464.1 eV, attributable to Ti 2p_3/2_ and Ti 2p_1/2_, respectively, of Ti^4+^ in TiO_2_. While the binding energy of Ti 2p for MT1.0 shifted to lower binding energy compared to MT3.0, which may be ascribed to the change of chemical environment for Ti, due to the interaction of PEI with TiO_2_.^50^ The O 1s spectra (Figure 2f) for MT1.0 and MT3.0 showed a stronger peak at 529.7 eV with a broader shoulder at 531.7 eV, which were ascribed to lattice oxygen in TiO_2_ and bridging hydroxyls, respectively.^51^ Furthermore, the C 1s spectra (Figure S10b, Supporting Information) revealed the presence of two types of C bonds. The strong peak at 284.6 eV corresponded to adventitious elemental C, and the other two peaks at 285.8 and 288.4 eV were characteristic of carbonates.^52^ The N 1s spectra (Figure S10c, Supporting Information) exhibited three peaks with binding energies of 398.8, 399.4, and 400.8 eV, which matched well the characteristic peaks of primary amino, secondary amino, and tertiary amino groups, respectively.^53^

Based on the above results, we came up with a polymer‐oriented self‐assembly strategy for the synthesis of mesoporous TiO_2_ with ultrahigh surface areas. This approach enabled a high degree of control over structural parameters, crystallinity, and morphology. As shown in Scheme 1, at the first stage, TBOT was added into H_2_O/PEI/HOAc solution, titanium oligomers hydrolyzed from TBOT could associate with PEI molecules through electrostatic interaction, giving rise to a white colloidal suspension of tiny titanium/PEI composites. When the amount of HOAc was less than 1.5 mL (MT1.0), TiO_2_ was negatively charged because the pH_r_ (about 7.0) was higher than pH_i.e_ of TiO_2_ (about 4.5). PEI was positively charged because portions of amine were protonated. Driven by Coulomb force, the positively charged PEI (S^+^) molecules captured anionic titanium oligomers (I^−^) and located in the interstitial space of titanium oligomers to play a role of porogen on mesoscale through electrostatic interaction (S^+^ I^−^) and thus initiated organic–inorganic assembly into mesoporous TiO_2_/PEI composites. By the subsequent hydrothermal treatment at 100 °C, the cross‐linking and condensation of titanium oligomers was further improved. By washing with distilled water and absolute ethanol several times, most of PEI molecules were dissolved, leaving behind a large amount of mesopores. However, there were still many of PEI molecules residues in mesoporous TiO_2_. Owing to strong electrostatic interaction between titanium species and PEI molecules, the presence of PEI molecules were incorporated into the structure of mesoporous TiO_2_, which contributed to maintaining the mesostructured stability, allowing formation of ultrahigh surface area mesoporous TiO_2_. When the amount of HOAc was more than 3 mL (MT3.0), the surface charges of titanium species have been transformed from negative to positive because the pH_r_ (about 4.0) was lower than pH_i.e_ of TiO_2_. Acetate groups could bind to titanium species through bridging modes and interact with PEI molecules by hydrogen‐bonding interaction. Therefore, acetate groups served as a glue which bound PEI molecules around titanium species through organic–inorganic interaction mode (S^+^ X^−^ I^+^). Compared to strong electrostatic interaction (S^+^ I^−^), this interaction between titanium species and PEI was relatively weaker. After hydrothermal treatment, S^+^ X^−^ I^+^ was not strong enough to restrict the growth of TiO_2_ nanocrystals under high temperature and high pressure environment, resulting in highly crystallized mesoporous TiO_2_.

**Figure 3:**
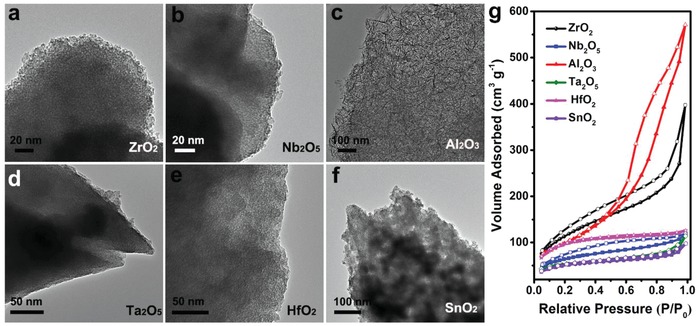
a–f) TEM images and g) nitrogen sorption isotherms of MMOs.

Under the guidance of mechanism studies, the polymer‐oriented self‐assembly strategy can be extended to synthesize other types of MMOs with ultrahigh surface areas, including ZrO_2_, NbO_5_, Al_2_O_3_, Ta_2_O_5_, HfO_2_, and SnO_2_. A large number of mesopores with uniform open pores of these MMOs can be clearly observed from TEM images (**Figure** **3**a–f). XRD patterns indicated that most MMOs except for mesoporous Al_2_O_3_ were amorphous at atomic scale (Figure S11, Supporting Information). Crystalline Al_2_O_3_ consisting of agglomerated nanoflakes was obtained after calcining mesoporous AlOOH at 400 °C in air (Figure S12a–d, Supporting Information). Nitrogen sorption data (Figure 3g; Figure S13, Supporting Information) indicated that all these samples possessed ultrahigh surface areas, uniform pore size distributions, and large pore volumes. The *S*_BET_ of these samples were as high as 422 m^2^ g^−1^ for ZrO_2_, 360 m^2^ g^−1^ for Nb_2_O_5_, 360 m^2^ g^−1^ for AlOOH, 393 m^2^ g^−1^ for Al_2_O_3_, 190 m^2^ g^−1^ for Ta_2_O_5_, 344 m^2^ g^−1^ for HfO_2_, and 174 m^2^ g^−1^ for SnO_2_ (Table 1).

MMOs can also be synthesized by using hydrochloric acid (HCl) as a pH regulator, and the corresponding results were shown in Figure S14a–g and Table S4 (Supporting Information). The resulting mesoporous TiO_2_ had a surface area of 550 m^2^ g^−1^, which was lower than that of MT1.0 (733 m^2^ g^−1^). This may be attributed to the complexation of HOAc that can modify the condensation kinetics of metal alkoxides. Moreover, under alkaline condition, our approach was still suitable for the synthesis of MMOs (Figure S15 and Table S5, Supporting Information). More importantly, we found that other nonsurfactant cationic polymers, such as PDADMAc, was also capable of directing the synthesis of MMOs (Figure S16 and Table S6, Supporting Information). These results confirmed that our synthesis system was very robust.

**Figure 4:**
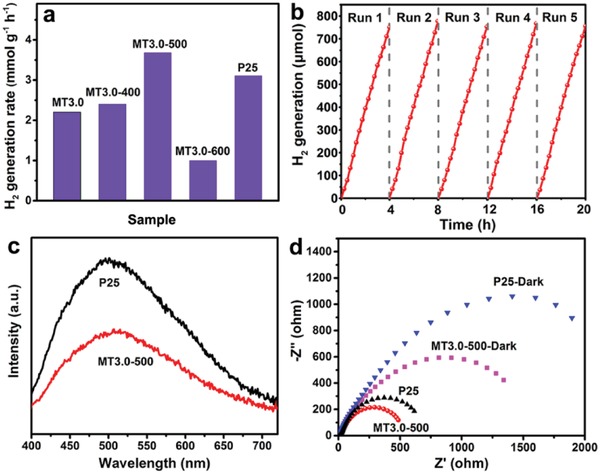
a) Comparisons of average photocatalytic H_2_ generation rates of the five TiO_2_ photocatalysts, 50 mg of photocatalysts were loaded with 1 wt% Au in 25 vol% methanol aqueous solution. b) Cycling tests of photocatalytic H_2_ generation of MT3.0 calcined at 500 °C as a function of UV–vis light irradiation time. c) Photoluminescence spectra of MT3.0 calcined at 500 °C and commercial P25 TiO_2_ under 375 nm laser excitation. d) Nyquist plots of electrochemical impedance of MT3.0 calcined at 500 °C and commercial P25 TiO_2_ under dark and simulated AM 1.5.

Photocatalytic water splitting for hydrogen production is one of the key technologies for addressing global energy problem.^54^ TiO_2_, one of the most important oxide semiconductors, has been proven to be a promising photocatalyst for photocatalytic hydrogen production, owing to its cheap price, abundance, low nontoxicity, and good stability.^55^, ^56^, ^57^ As part of this effort, we performed H_2_ evolution experiment from water splitting to evaluate the photocatalytic activity of as‐prepared mesoporous TiO_2_ (1 wt% Au loaded) with CH_3_OH as the sacrificial agent under a 300 W xenon lamp irradiation. With increasing the calcination temperature from 400 to 600 °C, the crystallinity of MT3.0 gradually improved while the corresponding *S*_BET_ decreased from 152 to 40 m^2^ g^−1^ (Figure S17a–c and Table S7, Supporting Information). **Figure** **4**a showed the temperature‐dependent photocatalytic H_2_ generation rates using obtained MT3.0 sample and commercial P25 TiO_2_ as photocatalysts. The H_2_ evolution rates increased with increasing the calcination temperature and reached a maximum at 500 °C, then decreased with further increasing the calcination temperature. The maximum H_2_ evolution rate of 3.68 mmol h^−1^ g^−1^ for MT3.0‐500 (MT3.0 calcined at 500 °C) was higher than that of commercial P25 TiO_2_ (3.09 mmol h^−1^ g^−1^), which was attributed to the large surface area and high crystallinity of MT3.0‐500. The *S*_BET_ of MT3.0‐500 (101 m^2^ g^−1^) was much larger than that of P25 TiO_2_ (50 m^2^ g^−1^). In general, the large surface area not only accommodated more catalysis active sites and accelerated the surface reaction kinetics, but also facilitated the transportation of reactant and product ions/molecules, which was responsible for photocatalytic activity.^58^ Furthermore, high crystallinity generally meant less defects and would be beneficial for the transfer and separation of photogenerated carriers.^59^ Thus, the photocatalytic activity of MT3.0‐500 was higher than that of MT3.0‐400. The photocatalytic activity of MT3.0‐600 was lower than that of MT3.0‐500, which was attributed to the occurrence of phase change and low surface area of MT3.0‐600. Moreover, the temperature‐dependent photocatalytic activities of MT1.0 were also investigated and the corresponding results were shown in Figure S18 and Table S8 of the Supporting Information. The MT1.0‐400 exhibited a much higher photocatalytic H_2_ generation rate of 4.49 mmol h^−1^ g^−1^ than those of other photocatalysts. Generally, stability was a crucial factor for photocatalysts in practical applications. To show its long‐term durability and reusability, the photocatalyst was reused for photocatalytic H_2_ production in 5 cycling tests within 20 h photocatalytic period. As shown in Figure 4b, no noticeable decrease in H_2_ production rate was observed, which indicated the photocatalyst possessed excellent stability.

To confirm the great influence of material structure on photocatalytic activity, photoluminescence (PL) spectra were performed, which is a very useful technique to disclose the photophysical processes of semiconductors, such as charge‐transfer and separation. From PL spectra shown in Figure 4c, the lower emission intensity of MT3.0‐500 than that of P25 TiO_2_ was observed clearly, indicating a more efficient charge separation. The electrochemical impedance measurement was employed to further clarify the photogenerated charge transfer efficiency. As shown in Figure 4d, a smaller interfacial resistance for MT3.0‐500 than that P25 TiO_2_ was observed, suggesting a higher separated efficiency of photogenerated electron–hole pairs. These results may contribute to the high photocatalytic activity of MT3.0‐500.

## 3 Conclusion

In summary, a facile and general polymer‐oriented self‐assembly strategy, using water as a solvent and nonsurfactant cationic polymers as porogens, for the fast and scalable synthesis of a series of MMOs (e.g., TiO_2_, ZrO_2_, NbO_5_, Al_2_O_3_, Ta_2_O_5_, HfO_2_, and SnO_2_) with ultrahigh specific surface areas and large pore volumes was developed. The presented strategy overcomes the defects associated with the traditional sol–gel‐based self‐assembly route in organic solvents synthesis system, which would strongly promote the industrial application of MMOs. These obtained MMOs possessing superior porous properties will be tailored for promising applications in energy conversion, catalyst, gas separation, and sensing. This approach may open up new opportunities for fabricating other high surface area mesoporous materials, such as mixed‐metal oxides, metal phosphates, metal sulfides, and oxide nanostructures.

## 4 Experimental Section

*Materials*: TBOT, zirconiumn butoxide (Zr(OBu)_4_), hafnium ethoxide, niobium ethoxide, tantalum ethoxide, aluminum ethoxide, tin(II)ethoxide, PEI (average Mw 600), and PDADMAc were purchased from Sigma‐Aldrich Co. HOAc concentrated HCl and ammonium hydroxide (NH_3_·H_2_O) were of analytical grade and obtained from Shanghai Chemical Corp.

*Synthesis of MMOs*: All the MMOs were synthesized by the polymer‐oriented self‐assembly strategy using nonsurfactant cationic polymers as porogens and metal alkoxides as metal oxide precursors, certain quantity HOAc as the pH regulator and complexing agent, and distilled water as a solvent. A typical synthesis of mesoporous TiO_2_, 3.4 mL TBOT, 1.6 g PEI, and a certain quantity HOAc were added in 30 mL distilled water. After stirring for 2 h, the obtained colloidal suspensions were transferred into an 80 mL autoclave and reacted at 100 °C for 24 h. The resulting powders were washed and then dried at 75 °C for 12 h. In order to investigate the effect of HOAc content on the pore structure and crystallinity of mesoporous TiO_2_, the HOAc content verified from 0 to 8 mL in the reaction system.

The synthesis of other MMOs, except for mesoporous Al_2_O_3_, was completed identically to that outlined for mesoporous TiO_2_. For the synthesis of mesoporous Al_2_O_3_, 10 mmol Al(C_2_H_5_O)_3_, 1 mL HOAc, 1.6 g PEI were dissolved in 30 mL distilled water. After stirring for 2 h, the obtained colloidal suspensions were transferred into an 80 mL autoclave and reacted at 100 °C for 24 h. The resulting powders were washed and then dried at 75 °C for 12 h. The obtained mesoporous AlOOH was calcined at 400 °C in air for 5 h (ramp rate 2 °C min^−1^) to obtain mesoporous Al_2_O_3_.

*Photocatalytic Activity Measurements*: The photocatalytic water splitting was carried out in an online photocatalytic hydrogen generation system (CEL‐SPH2N‐D9, Beijing Aulight Co., Ltd.) at ambient temperature. 50 mg of photocatalyst powder loaded with 1 wt% Au was dispersed in 50 mL of aqueous solution containing 10 mL of methanol and 40 mL of water. Prior to the 300 W xenon lamp irradiation, the mixture was evacuated for 30 min to remove oxygen and ensure that the reactor was in a vacuum condition. The resultant hydrogen was determined by an on‐line gas chromatograph (GC7920‐DTA) equipped with a TCD detector.

*Photoelectrochemical Property*: The electrochemical impedance measurements were performed using a three‐electrode CHI 650D electrochemical workstation in a 0.5 m Na_2_SO_4_ electrolyte, with Pt foil as counter electrode, saturated calomel electrode as reference electrode, and photocatalysts used as working electrodes. A simulated AM 1.5 solar power system was used as light irradiation source. The working electrodes were prepared by dropping the suspension containing 20 mg of powder and 20 µL of absolute ethanol on F‐doped tin oxide glass (0.5 cm × 0.5 cm).

*Characterizations*: XRD patterns were measured using a Bruker D8 Advance X‐ray diffractometer with monochromatic Cu Kα irradiation. N_2_ sorption isotherms were measured at 77 K with a Quantachrome Nova 4200e. Before measurements, all of the samples were degassed under vacuum at 100 °C for a minimum of 8 h. The specific surface area was calculated by BET equation. Pore size distributions were obtained using the nonlocal density function theory method from the adsorption branch of the isotherms. FT‐IR spectra were recorded on a Perkin‐Elmer 580B infrared spectrophotometer. XPS measurements were measured on an ESCALab220i‐XL electron spectrometer. TEM analysis was performed on FEI Tenia G2 F20 microscope operated at 200 kV. SEM analysis was conducted on JEOL JSM‐6700F field‐emission scanning electron microscope operated under 15 kV. Photoluminescence emission spectra (375 nm excitation) were performed with a Hitachi F‐7000 spectrophotometer. The weight loss of the samples was measured using a thermogravimetric analyzer (TGA Q50) in air with a heating rate of 10 °C min^−1^. Raman spectra were performed on a Renishaw via Raman microscope.

## Conflict of Interest

The authors declare no conflict of interest.

## Supporting information

Supplementary

## References

[1] W. Li , J. Liu , D. Zhao , Nat. Rev. Mater. 2016, 1, 16023.

[2] Y. Ren , Z. Ma , P. G. Bruce , Chem. Soc. Rev. 2012, 41, 4909. 22653082 10.1039/c2cs35086f

[3] H. Wang , S. Zhuo , Y. Liang , X. Han , B. Zhang , Angew. Chem., Int. Ed. 2016, 55, 9055. 10.1002/anie.201603197 27239778

[4] Y. Lyu , S. H. Yi , J. K. Shon , S. Chang , L. S. Pu , S. Lee , J. E. Yie , K. Char , G. D. Stucky , J. M. Kim , J. Am. Chem. Soc. 2004, 8, 126. 10.1021/ja0390348 14982427

[5] Y. Ma , Y. Zhang , X. Wang , M. Fan , K. Li , T. Wang , Y. Liu , Q. Huo , Z. A. Qiao , S. Dai , Nanoscale 2018, 10, 5731. 29537010 10.1039/c7nr07883h

[6] Q. Zhang , J. Wang , J. Dong , F. Ding , X. Li , B. Zhang , S. Yang , K. Zhang , Nano. Energy 2015, 13, 77.

[7] C. Jo , J. Hwang , W. Lim , J. Lim , K. Hur , J. Lee , Adv. Mater. 2018, 30, 1703829. 10.1002/adma.201703829 29271508

[8] Y. Oka , Y. Kuroda , T. Matsuno , K. Kamata , H. Wada , A. Shimojima , K. Kuroda , Chem. ‐ Eur. J. 2017, 23, 9362. 28514015 10.1002/chem.201701282

[9] I. E. Rauda , R. Buonsanti , L. C. Saldarriaga‐Lopez , K. Benjauthrit , L. T. Schelhas , M. Stefik , V. Augustyn , J. Ko , B. Dunn , U. Wiesner , D. J. Milliron , S. H. Tolbert , ACS Nano 2012, 6, 6386. 22731824 10.1021/nn302789r

[10] J. Lee , M. C. Orilall , S. C. Warren , M. Kamperman , F. J. DiSalvo , U. Wiesner , Nat. Mater. 2008, 7, 222. 18223653 10.1038/nmat2111

[11] B. P. Bastakoti , S. Ishihara , S. Leo , K. Ariga , K. C. Wu , Y. Yamauchi , Langmuir 2014, 30, 651. 24392806 10.1021/la403901x

[12] T. Froschl , U. Hormann , P. Kubiak , G. Kucerova , M. Pfanzelt , C. K. Weiss , R. J. Behm , N. Husing , U. Kaiser , K. Landfester , M. Wohlfahrt‐Mehrens , Chem. Soc. Rev. 2012, 41, 5313. 22763865 10.1039/c2cs35013k

[13] X. Deng , K. Chen , H. Tüysüz , Chem. Mater. 2016, 29, 40.

[14] W. Xiao , S. Yang , P. Zhang , P. Li , P. Wu , M. Li , N. Chen , K. Jie , C. Huang , N. Zhang , S. Dai , Chem. Mater. 2018, 30, 2924.

[15] W. Luc , F. Jiao , Acc. Chem. Res. 2016, 49, 1351. 27294847 10.1021/acs.accounts.6b00109

[16] X. Sun , Y. Shi , P. Zhang , C. Zheng , X. Zheng , F. Zhang , Y. Zhang , N. Guan , D. Zhao , G. D. Stucky , J. Am. Chem. Soc. 2011, 133, 14542. 21861449 10.1021/ja2060512

[17] E. Pellicer , M. Cabo , E. Rossinyol , P. Solsona , S. Suriñach , M. D. Baró , J. Sort , Adv. Funct. Mater. 2013, 23, 900.

[18] J. Smått , F. M. Sayler , A. J. Grano , M. G. Bakker , Adv. Eng. Mater. 2012, 14, 1059.

[19] H. Yen , Y. Seo , R. Guillet‐Nicolas , S. Kaliaguine , F. Kleitz , Chem. Commun. 2011, 47, 10473. 10.1039/c1cc13867g 21858306

[20] F. Jiao , P. G. Bruce , Adv. Mater. 2007, 19, 657.

[21] J. Deng , L. Zhang , H. Dai , Y. Xia , H. Jiang , H. Zhang , H. He , J. Phys. Chem. C 2010, 114, 2694.

[22] Y. Zhu , Y. Zhao , J. Ma , X. Cheng , J. Xie , P. Xu , H. Liu , H. Liu , H. Zhang , M. Wu , A. A. Elzatahry , A. Alghamdi , Y. Deng , D. Zhao , J. Am. Chem. Soc. 2017, 139, 10365. 28683546 10.1021/jacs.7b04221

[23] J. Liu , S. Zou , S. Li , X. Liao , Y. Hong , L. Xiao , J. Fan , J. Mater. Chem. A 2013, 1, 4038.

[24] S. W. Robbins , P. A. Beaucage , H. Sai , K. W. Tan , J. G. Werner , J. P. Sethna , F. J. DiSalvo , S. M. Gruner , R. B. V. Dover , U. Wiesner , Sci. Adv. 2016, 2, e1501119. 27152327 10.1126/sciadv.1501119 PMC4846463

[25] T. Brezesinski , J. Wang , S. H. Tolbert , B. Dunn , Nat. Mater. 2010, 9, 146. 20062048 10.1038/nmat2612

[26] Y. Liu , R. Che , G. Chen , J. Fan , Z. Sun , Z. Wu , M. Wang , B. Li , J. Wei , Y. Wei , G. Wang , G. Guan , A. A. Elzatahry , A. A. Bagabas , A. M. Al‐Enizi , Y. Deng , H. Peng , D. Zhao , Sci. Adv. 2015, 1, e1500166. 26601185 10.1126/sciadv.1500166 PMC4640639

[27] J. Wei , H. Wang , Y. Deng , Z. Sun , L. Shi , B. Tu , M. Luqman , D. Zhao , J. Am. Chem. Soc. 2011, 133, 20369. 22047467 10.1021/ja207525e

[28] Y. Zhang , X. Zhao , Y. Wang , L. Zhou , J. Zhang , J. Wang , A. Wang , T. Zhang , J. Mater. Chem. A 2013, 1, 3724.

[29] C. K. Tsung , J. Fan , N. Zheng , Q. Shi , A. J. Forman , J. Wang , G. D. Stucky , Angew. Chem., Int. Ed. 2008, 47, 8682. 10.1002/anie.200802487 18846512

[30] W. Zhou , W. Li , J. Q. Wang , Y. Qu , Y. Yang , Y. Xie , K. Zhang , L. Wang , H. Fu , D. Zhao , J. Am. Chem. Soc. 2014, 136, 9280. 24937035 10.1021/ja504802q

[31] W. Luo , Y. Li , J. Dong , J. Wei , J. Xu , Y. Deng , D. Zhao , Angew. Chem., Int. Ed. 2013, 52, 10505. 10.1002/anie.201303353 23943495

[32] Y. Ren , X. Zhou , W. Luo , P. Xu , Y. Zhu , X. Li , X. Cheng , Y. Deng , D. Zhao , Chem. Mater. 2016, 28, 7997.

[33] Y. Wan , H. Yang , D. Zhao , Acc. Chem. Res. 2006, 39, 423. 16846206 10.1021/ar050091a

[34] S. W. Boettcher , J. Fan , C. Tsung , Q. Shi , G. D. Stucky , Acc. Chem. Res. 2007, 40, 784. 17461540 10.1021/ar6000389

[35] J. Wei , Z. Sun , W. Luo , Y. Li , A. A. Elzatahry , A. M. Al‐Enizi , Y. Deng , D. Zhao , J. Am. Chem. Soc. 2017, 139, 1706. 28085258 10.1021/jacs.6b11411

[36] W. Li , Q. Yue , Y. Deng , D. Zhao , Adv. Mater. 2013, 25, 5129. 23868196 10.1002/adma.201302184

[37] J. Fan , S. W. Boettcher , G. D. Stucky , Chem. Mater. 2006, 18, 6391.

[38] A. S. Poyraz , C. H. Kuo , S. Biswas , C. K. King'ondu , S. L. Suib , Nat. Commun. 2013, 4, 2952. 24335918 10.1038/ncomms3952

[39] A. A. Soler‐Illia , E. L. Crepaldi , H. Amenitsch , A. Brunet‐Bruneau , A. Bourgeois , C. Sanchez , Adv. Funct. Mater. 2004, 14, 309.

[40] G. J. A. A. Soler‐Illia , C. Sanchez , New. J. Chem. 2000, 24, 493.

[41] E. L. Crepaldi , G. J. A. A. Soler‐Illia , D. Grosso , F. Cagnol , F. Ribot , C. Sanchez , J. Am. Chem. Soc. 2003, 125, 9770. 12904043 10.1021/ja030070g

[42] Y. Li , S. Wang , D. Lei , Y. He , B. Li , F. Kang , J. Mater. Chem. A 2017, 5, 12236.

[43] T. Ohsaka , F. Izumi , Y. Fujiki , J. Raman Spectrosc. 1978, 7, 321.

[44] L. Dang , G. Zhang , K. Kan , Y. Lin , F. Bai , L. Jing , P. Shen , L. Li , K. Shi , J. Mater. Chem. A 2014, 2, 4558.

[45] Y. Hu , C. Guo , F. Wang , S. Wang , F. Pan , C. Liu , Chem. Eng. J. 2014, 242, 341.

[46] A. Sayari , A. Heydari‐Gorji , Y. Yang , J. Am. Chem. Soc. 2012, 134, 13834. 22845036 10.1021/ja304888a

[47] J. Liao , B. Lei , D. Kuang , C. Su , Energy Environ. Sci. 2011, 4, 4079.

[48] S. W. Boettcher , M. H. Bartl , J. G. Hu , G. D. Stucky , J. Am. Chem. Soc. 2005, 127, 9721. 15998076 10.1021/ja050753r

[49] X. Xu , C. Song , J. M. Andresen , B. G. Miller , A. W. Scaroni , Energy Fuels 2002, 16, 1463.

[50] Y. Wang , S. Chen , X. Tang , O. Palchik , A. Zaban , Y. Koltypin , A. Gedanken , J. Mater. Chem. 2001, 11, 521.

[51] L. Li , J. Yan , T. Wang , Z. Zhao , J. Zhang , J. Gong , N. Guan , Nat. Commun. 2015, 6, 5881. 25562287 10.1038/ncomms6881

[52] B. Liu , L. Liu , X. Lang , H. Wang , X. Lou , E. S. Aydil , Energy Environ. Sci. 2014, 7, 2592.

[53] F. Zhao , E. Repo , Y. Song , D. Yin , S. Hammouda , L. Chen , S. Kalliola , J. Tang , K. Tam , M. Sillanpää , Green Chem. 2017, 19, 4816.

[54] N. S. Lewis , D. G. Nocera , Proc. Natl. Acad. Sci. USA 2006, 103, 15729. 17043226 10.1073/pnas.0603395103 PMC1635072

[55] X. Zhou , N. Liu , J. Schmidt , A. Kahnt , A. Osvet , S. Romeis , E. M. Zolnhofer , V. R. Marthala , D. M. Guldi , W. Peukert , M. Hartmann , K. Meyer , P. Schmuki , Adv. Mater. 2017, 29, 1604747. 10.1002/adma.201604747 27886413

[56] M. Li , Y. Chen , W. Li , X. Li , H. Tian , X. Wei , Z. Ren , G. Han , Small 2017, 13, 1604115. 10.1002/smll.201604115 28218501

[57] J. Zhang , Z. Yu , Z. Gao , H. Ge , S. Zhao , C. Chen , S. Chen , X. Tong , M. Wang , Z. Zheng , Y. Qin , Angew. Chem., Int. Ed. 2017, 56, 816. 10.1002/anie.201611137 27966808

[58] W. Zhou , F. Sun , K. Pan , G. Tian , B. Jiang , Z. Ren , C. Tian , H. Fu , Adv. Funct. Mater. 2011, 21, 1922.

[59] X. Chen , S. Shen , L. Guo , S. S. Mao , Chem. Rev. 2010, 110, 6503. 21062099 10.1021/cr1001645

