# Supplementary material for: A Polymer‐Oriented Self‐Assembly Strategy toward Mesoporous Metal Oxides with Ultrahigh Surface Areas
Source: Adv Sci (Weinh). 2019 Jan 28;6(6):1801543. doi: 10.1002/advs.201801543 (PMC6425444; doi:10.1002/advs.201801543)
Supplement: Supplementary file 1 — Supplementary [file ADVS-6-1801543-s001.pdf]

Copyright WILEY-VCH Verlag GmbH & Co. KGaA, 69469 Weinheim, Germany, 2019.

ADVANCED SCIENCE
Open Access

Supporting Information

for *Adv. Sci.,* DOI: 10.1002/advs.201801543

A Polymer-Oriented Self-Assembly Strategy toward Mesoporous Metal Oxides with Ultrahigh Surface Areas

Hailong Xiong, Tunan Gao, Kaiqian Li, Yali Liu, Yali Ma, Jingwei Liu, Zhen-An Qiao, Shuyan Song, and Sheng Dai*

Supplementary Information

A Polymer-oriented Self-assembly Strategy towards Mesoporous Metal Oxides with Ultrahigh Surface Areas

Hailong Xiong, Tunan Gao, Kaiqian Li, Yali Liu, Yali Ma, Jingwei. Liu, Zhen-An Qiao*, Shuyan Song, and Sheng Dai

H. Xiong, T. Gao, K. Li, Y. Liu, A. Li, Y. Ma, Prof. Z.-A. Qiao
State Key Laboratory of Inorganic Synthesis and Preparative Chemistry, Jilin University, Changchun, Jilin 130012 (China)
E-mail: qiaozhenan@jlu.edu.cn

Dr. S. Dai
Chemical Sciences Division, Oak Ridge National Laboratory,
Oak Ridge, TN, 37831, USA

Dr. S. Y. Song
Key Laboratory of Rare Earth Chemistry and Physics, Changchun Institute of Applied Chemistry, Graduate School of the Chinese Academy of Sciences, Chinese Academy of Sciences, Changchun, Jilin 130022 (China)

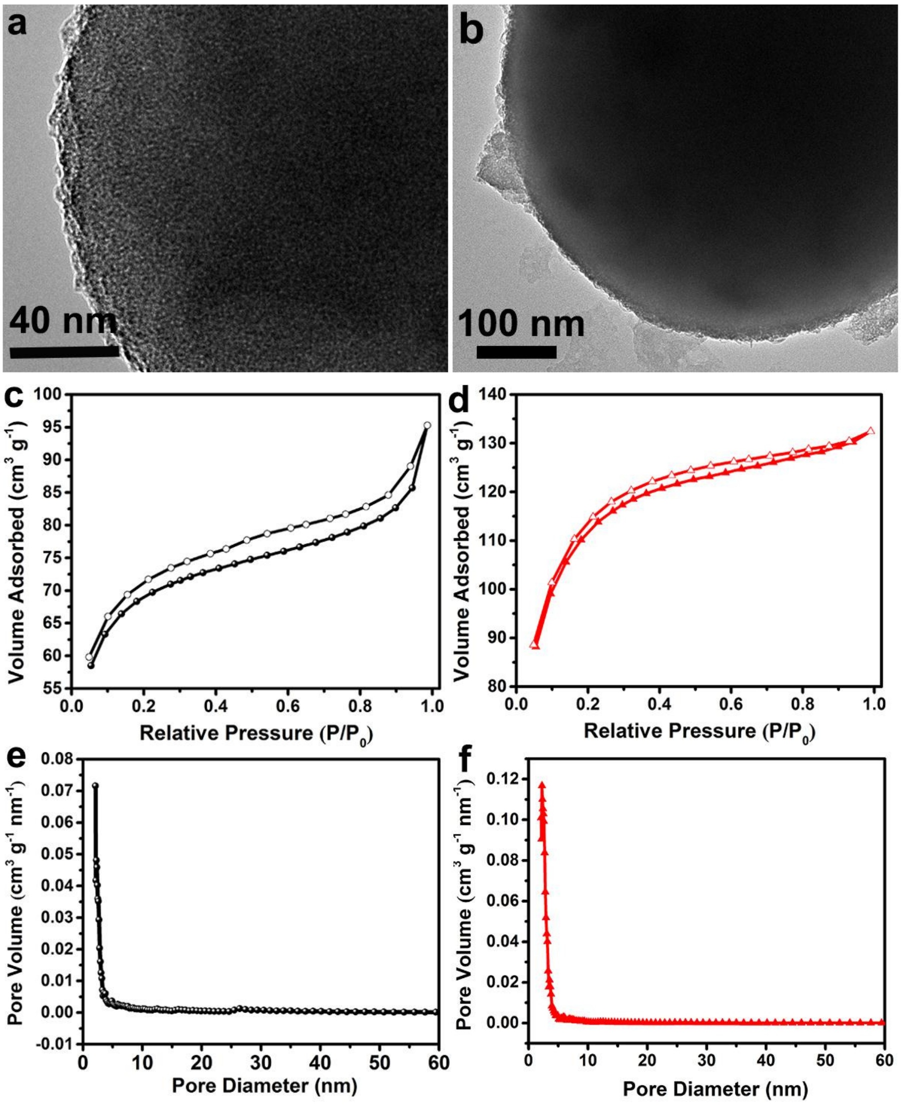

Figure S1. TEM image (a), nitrogen sorption isotherms (c), and pore size distribution (e) of mesoporous TiO_2 prepared at room temperature for 2 h with 1.0 mL HOAc. TEM image (b), nitrogen sorption isotherms (d), and pore size distribution (f) of mesoporous TiO_2 prepared at room temperature for 2 h with 3.0 mL HOAc.

Table S1 Structural properties of mesoporous TiO_2 prepared at room temperature for 2 h with different HOAc content.

HOAc content (mL)	S_{BET} [a] (m^2 g^{-1})	Pore size (nm)	V_T [b] (cm^3 g^{-1})
1.0	362	2.2	0.238
3.0	563	2.3	0.345

Note: [a] BET surface area and [b] total pore volume.

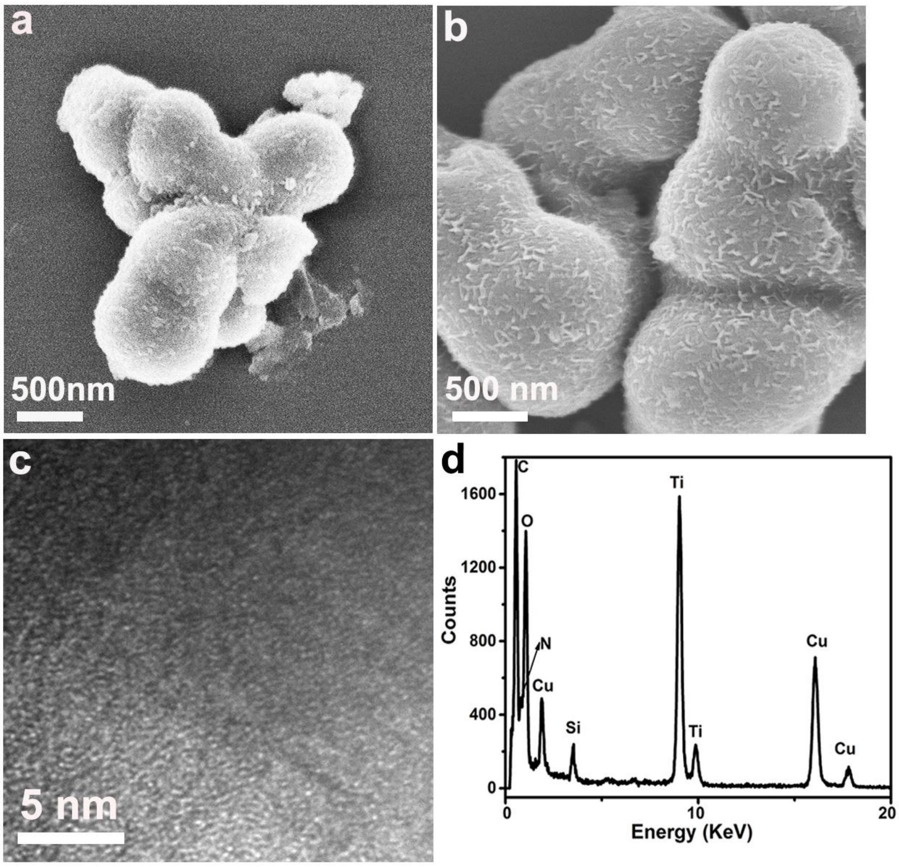

Figure S2. SEM image (a) of MT0. SEM image (b), HRTEM image (c) and EDX spectra (d) of MT1.0.

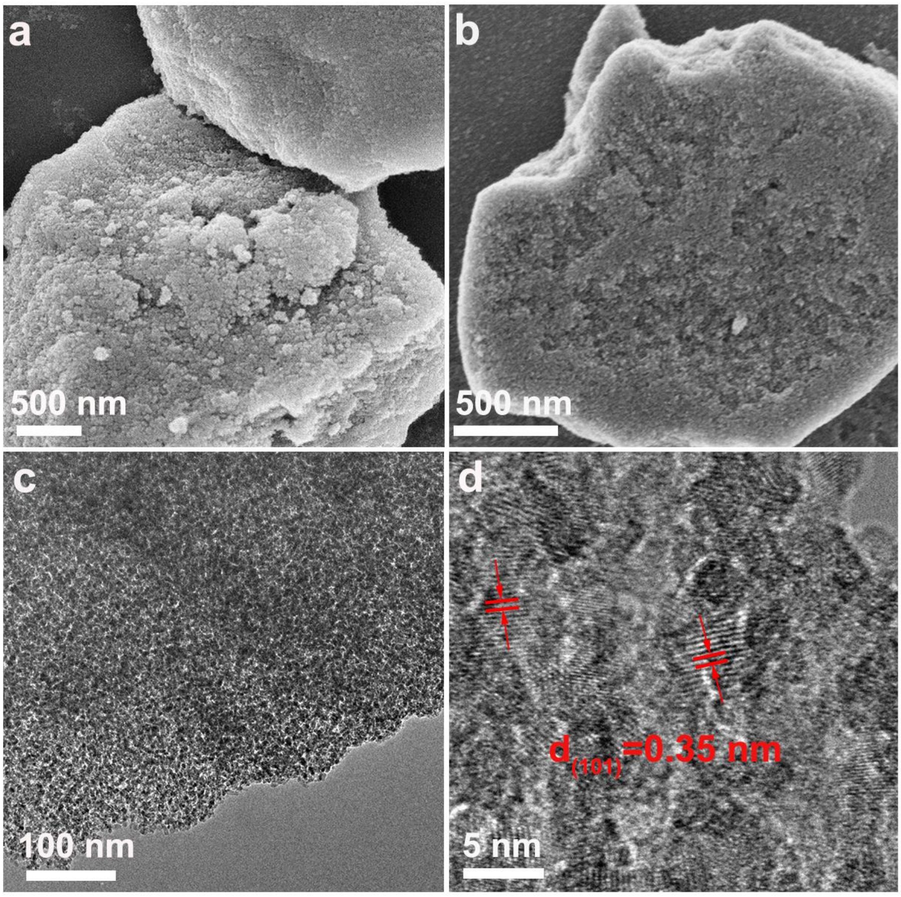

Figure S3. SEM images of MT2.5 (a) and MT3.0 (b). TEM image (c) and HRTEM image (d) of MT2.5.

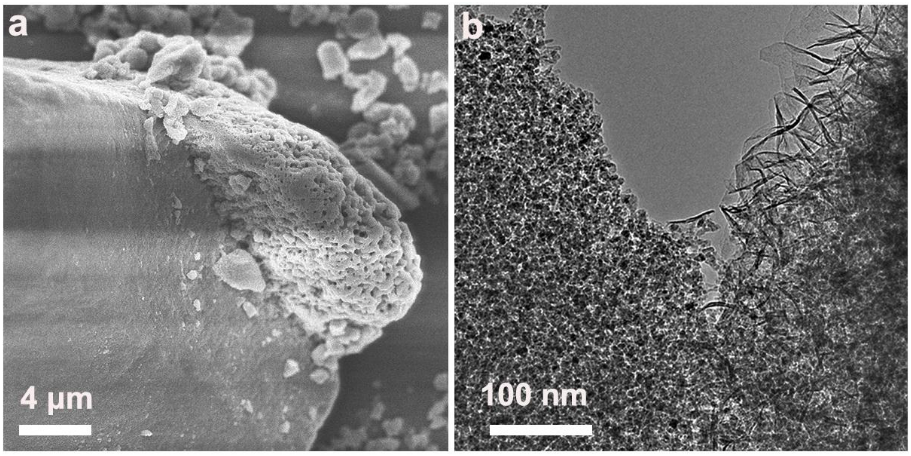

Figure S4. SEM image (e) and TEM image (f) of MT2.0.

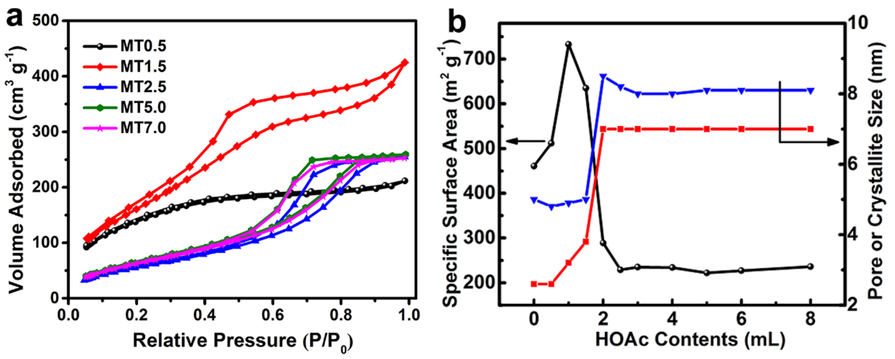

Figure S5. Nitrogen sorption isotherms (a) of mesoporous TiO_2: MT0.5, MT1.5, MT2.5, MT5.0, and MT7.0. (b) Specific surface area (black line), average pore size (red line), and average crystallite size (blue line) of mesoporous TiO_2 as a function of HOAc content from 0 mL to 8 mL.

Table S2 Structural properties of mesoporous TiO_2 prepared at 100 °C for 24 h with different HOAc content.

Sample	S_{BET} [a] (m^2 g^{-1})	Pore size (nm)	V_T [b] (cm^3 g^{-1})
MT0.5	512	2.6	0.327
MT1.5	635	3.8	0.657
MT2.5	229	7.0	0.395
MT4.0	234	7.0	0.401
MT5.0	222	7.0	0.398
MT6.0	227	7.0	0.389
MT7.0	228	7.0	0.392
MT8.0	236	7.0	0.410

Note: [a] BET surface area and [b] total pore volume.

Table S3 Comparison of the surface area of mesoporous TiO_2 synthesized by different methods.

Synthesis method	S_{BET} (m^2 g^{-1})	Ref.
Polymer-oriented self-assembly strategy	733	This work
Ligand-assisted assembly approach	112	Zhang et al. [S1]
Evaporation-induced self-assembly method	289	Zhou et al. [S2]
Noaqueous solvothermal method	500	Liu et al. [S3]
Double-surfactant assembly-directed method	160	Zhu et al. [S4]
Evaporation-induced self-assembly method	112	Liu et al. [S5]
Confined microemulsion self-assembly approach	148	Liu et al. [S6]
Pressure-driven oriented assembly approach	124	Lan et al. [S7]

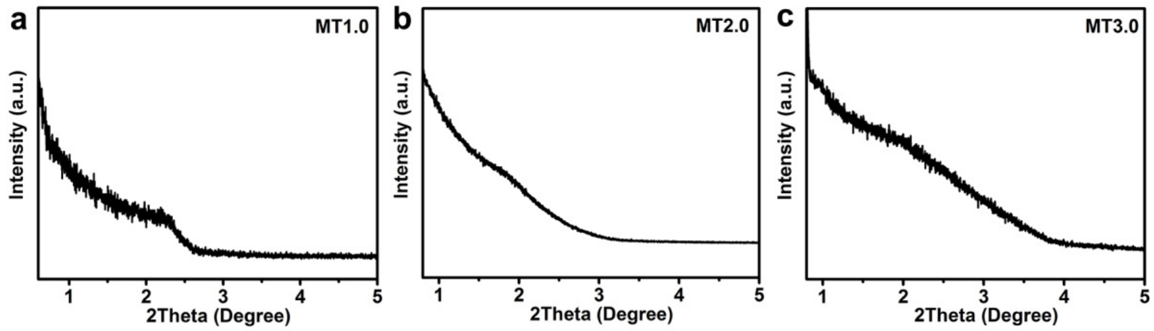

Figure S6. Small angle XRD patterns of mesoporous TiO_2: MT1.0, MT2.0 and MT3.0, respectively.

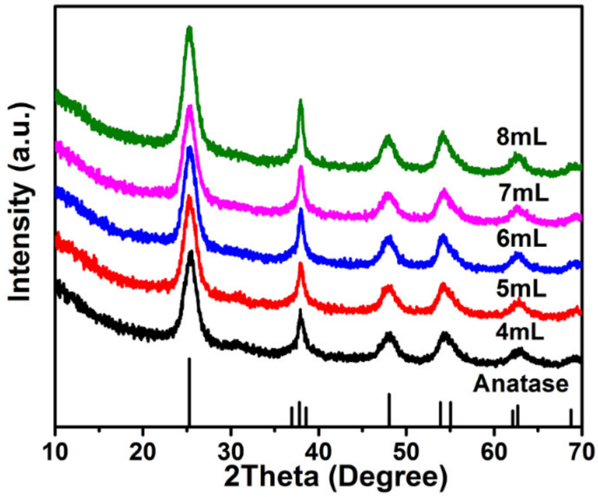

Figure S7. XRD patterns of mesoporous TiO_2 prepared at 100 °C for 24 h with different HOAc content.

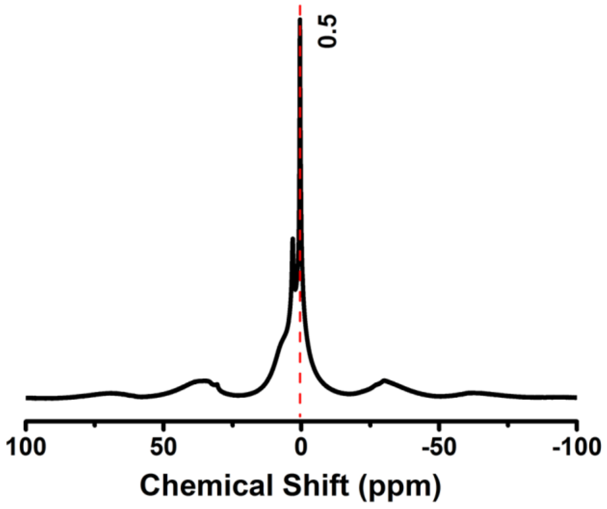

Figure S8. ^{1}H NMR spectrum of MT1.0.

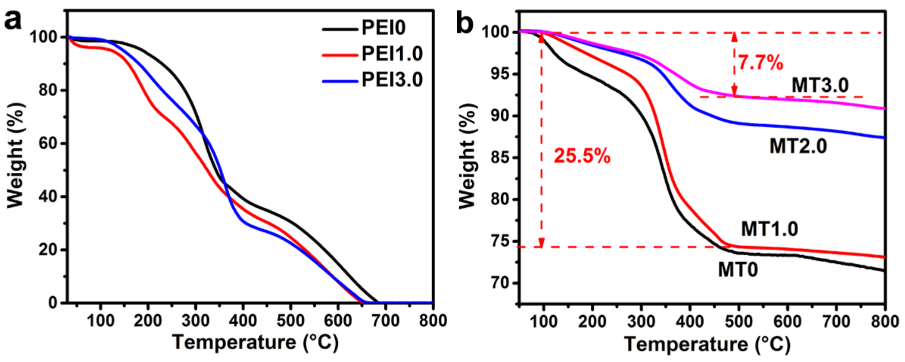

Figure S9. TG curves of PEIx after hydrothermal treatment (a) and MTx (b), x referred to the amount of HOAc.

As shown in Figure S8a, PEIx after hydrothermal treatment showed similar TG curves. The main weight loss of PEIx was in the range of 120 - 450 °C, attributed to the decomposition of PEI, which was in good agreement with the TG curves of MTx. In addition, broad weight loss above 450 °C is due to the combustion of the last organic fragments. The weight loss of MT0 and MT1.0 below 450 °C was much larger than that of MT2.0 and MT3.0 (Figure S8b), indicating more PEI molecules in the pores of MT0 and MT1.0. The possible reason can be explained as follows: When

the amount of HOAc was less than 1.5 mL, TiO_2 was negatively charged because the pH of reaction system (about 7.0) was higher than isoelectric point of TiO_2 (about 4.5). PEI was positively charged because portions of amine were protonated. Driven by Coulomb force, the positively charged PEI (S^+) molecules captured anionic titanium oligomers (I^-) through electrostatic interaction ($S^+ I^-$). When the amount of HOAc was larger than 3.0 mL, the pH of reaction system (about 4.0) was lower than isoelectric point of TiO_2. The positively charged PEI molecules could interact with cationic titanium oligomers through organic-inorganic interaction mode ($S^+ X^- I^+$). Compared to strong electrostatic interaction ($S^+ I^-$), the organic-inorganic interaction mode ($S^+ X^- I^+$) between titanium species and PEI was relatively weaker. Thus, there were more PEI molecules residues in MT0 and MT1.0 compared to MT2.0 and MT3.0 after hydrothermal treatment.

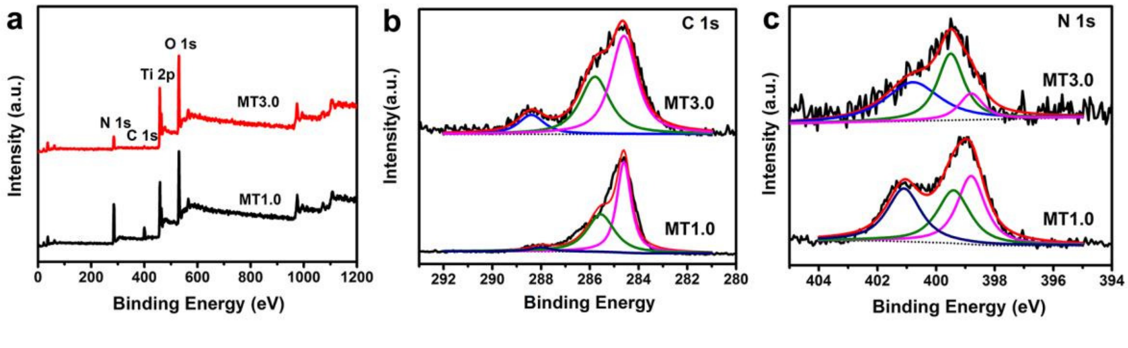

Figure S10. XPS survey spectra (a), C 1s (b), and N 1s (c) high-resolution XPS spectra of mesoporous TiO_2: MT1.0 and MT3.0, respectively.

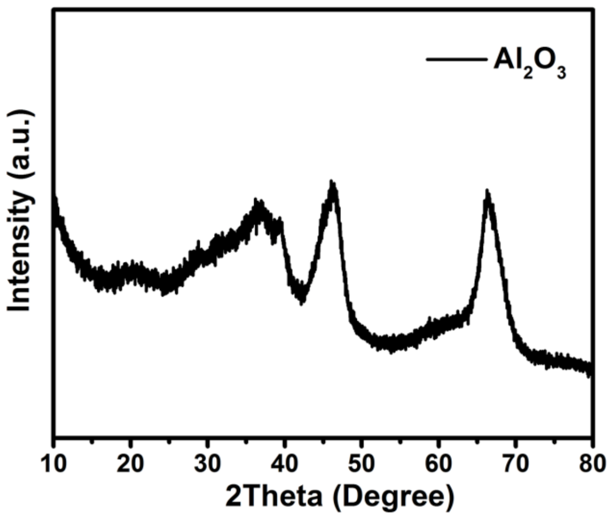

Figure S11. XRD pattern of mesoporous Al_2O_3 obtained after calcining the mesoporous AlOOH at 400 ^{o}C in air.

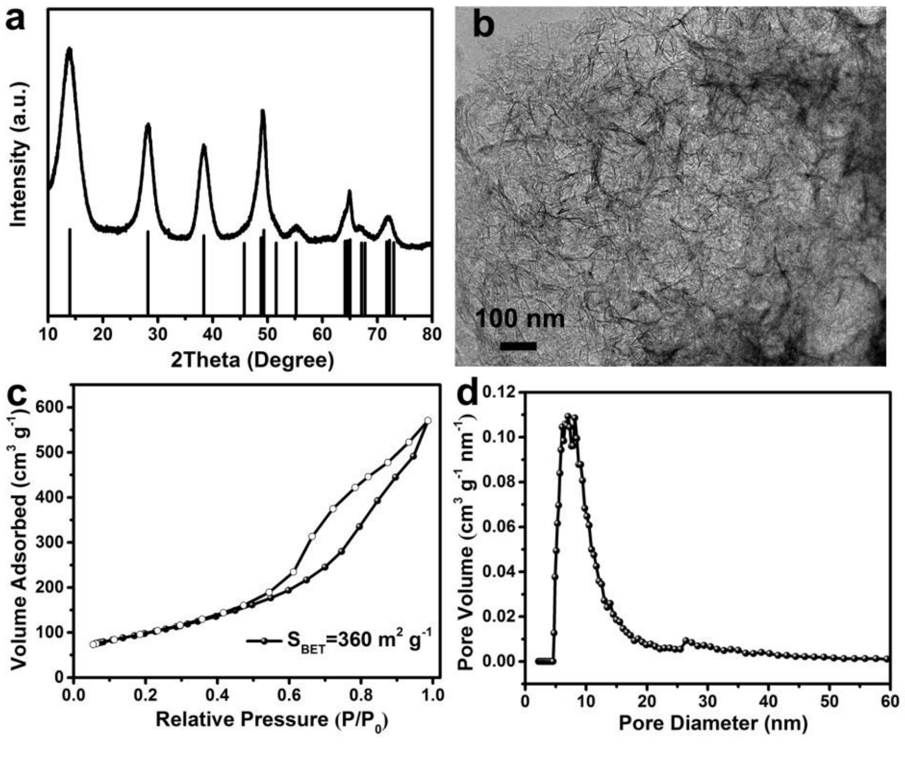

Figure S12. XRD pattern (a), TEM image (b), nitrogen sorption isotherms (c), and pore size distribution (d) of mesoporous AlOOH prepared at 100 °C for 24 h.

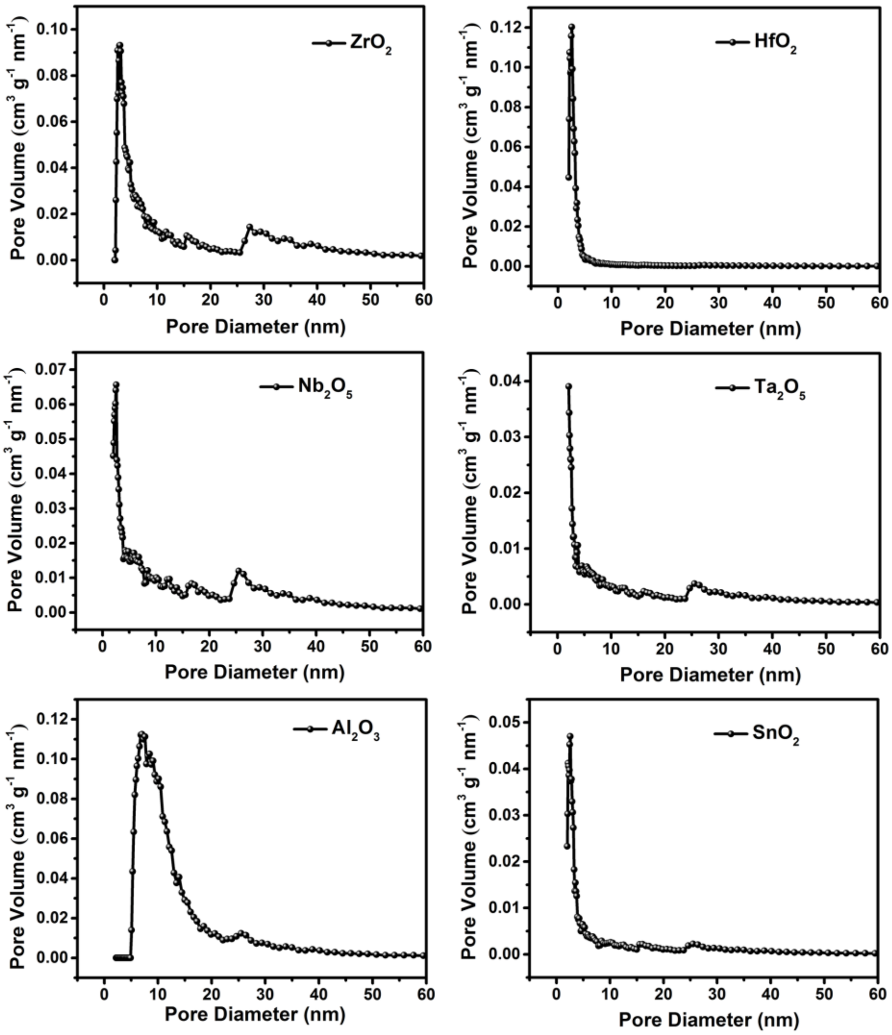

Figure S13. Pore size distributions of MMOs.

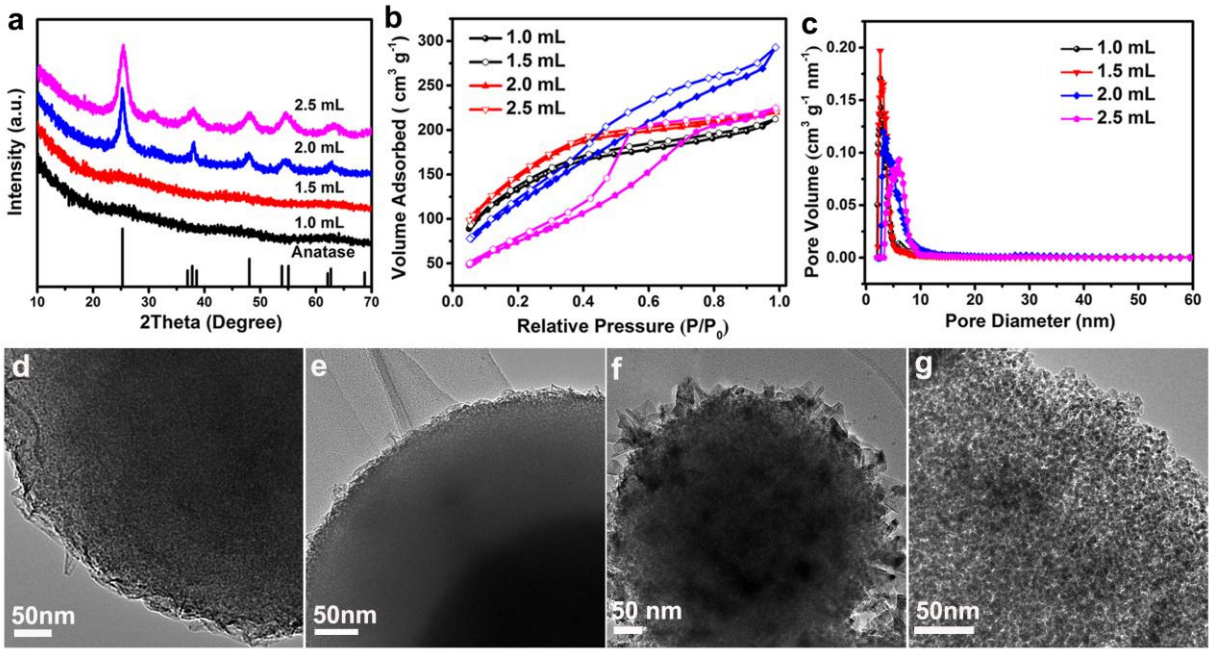

Figure S14. XRD patterns (a), nitrogen sorption isotherms (b), pore size distributions (c), and TEM images (d-g) of mesoporous TiO_2 prepared at 100 °C for 24 h with different HCl content: 1.0 mL (d), 1.5 mL (e), 2.0 mL (f), and 2.5 mL (g).

Table S4 Structural properties of mesoporous TiO_2 prepared at 100 °C for 24 h with different HCl content.

$NH_3{\cdot}H_2O$ content (mL)	S_{BET} [a] (m^2 g^{-1})	Pore size (nm)	V_T [b] (cm^3 g^{-1})
1.0	480	2.6	0.328
1.5	550	2.6	0.340
2.0	456	3.2	0.453
2.5	290	6.1	0.348

Note: [a] BET surface area and [b] total pore volume.

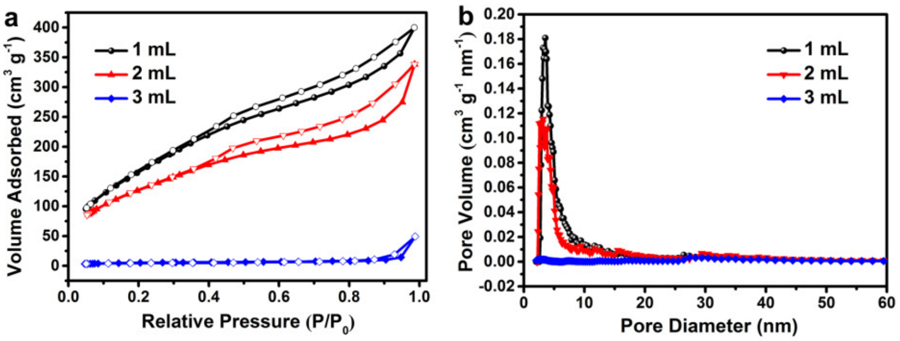

Figure S15. Nitrogen sorption isotherms (a) and pore size distributions (b) of mesoporous TiO_2 prepared at 100 °C for 24 h with different $NH_3{\cdot}H_2O$ content.

Table S5 Structural properties of mesoporous TiO_2 prepared at 100 °C with different $NH_3{\cdot}H_2O$ content.

$NH_3{\cdot}H_2O$ content (mL)	S_{BET} [a] (m^2 g^{-1})	Pore size (nm)	V_T [b] (cm^3 g^{-1})
1.0	623	3.5	0.619
2.0	479	3.2	0.523
3.0	18	-	-

Note: [a] BET surface area and [b] total pore volume.

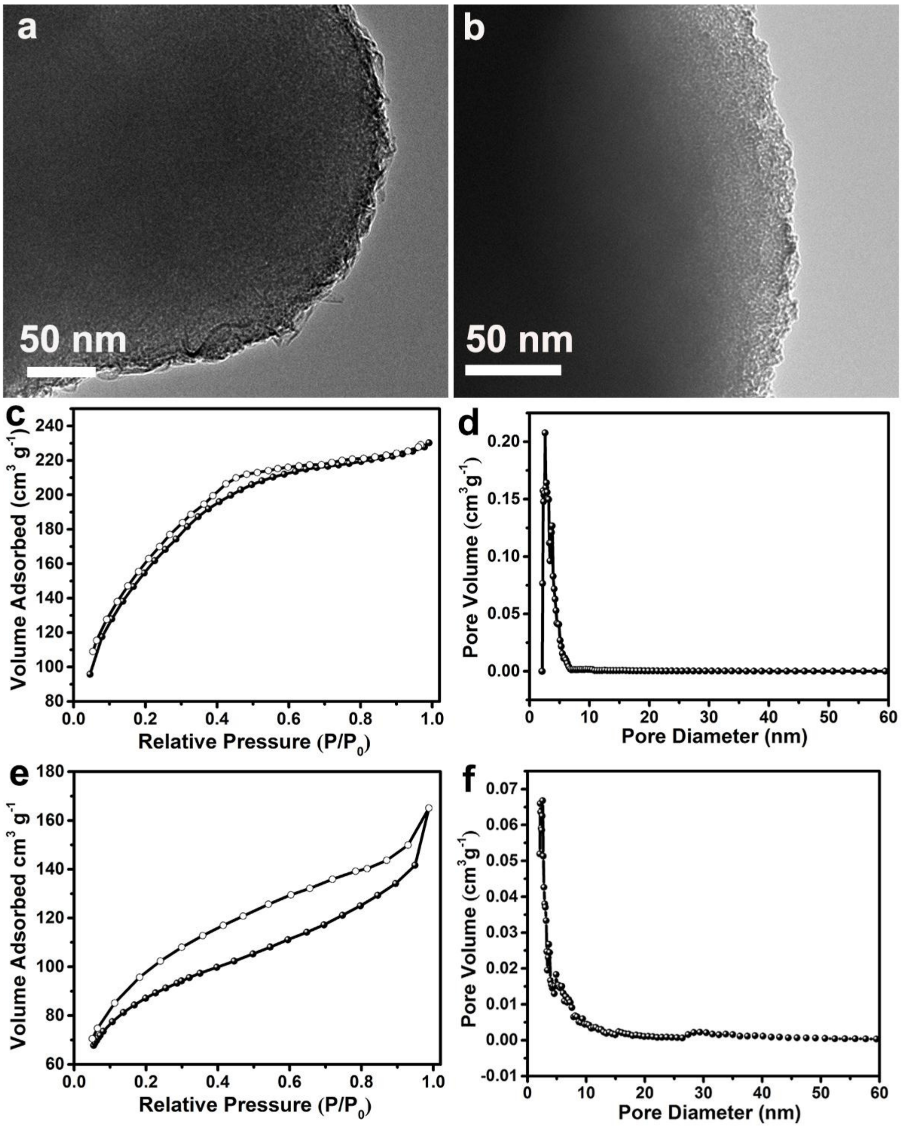

Figure S16. TEM image (a), nitrogen sorption isotherms (c), and pore size distributions (e) of mesoporous TiO_2 prepared at 100 °C for 24 h using PDADMAc as a porogen instead of PEI. TEM image (b), nitrogen sorption isotherms (d), and pore size distributions (f) of mesoporous ZrO_2 prepared at 100 °C for 24 h using PDADMAc as a porogen instead of PEI.

Table S6 Structural properties of MMOs prepared at 100 °C d for 24 h using PDADMAc as a porogen instead of PEI.

Sample	S_{BET} [a] (m^2 g^{-1})	Pore size (nm)	V_T [b] (cm^3 g^{-1})
TiO_2	575	2.6	0.335
ZrO_2	328	2.6	0.242

Note: [a] BET surface area and [b] total pore volume.

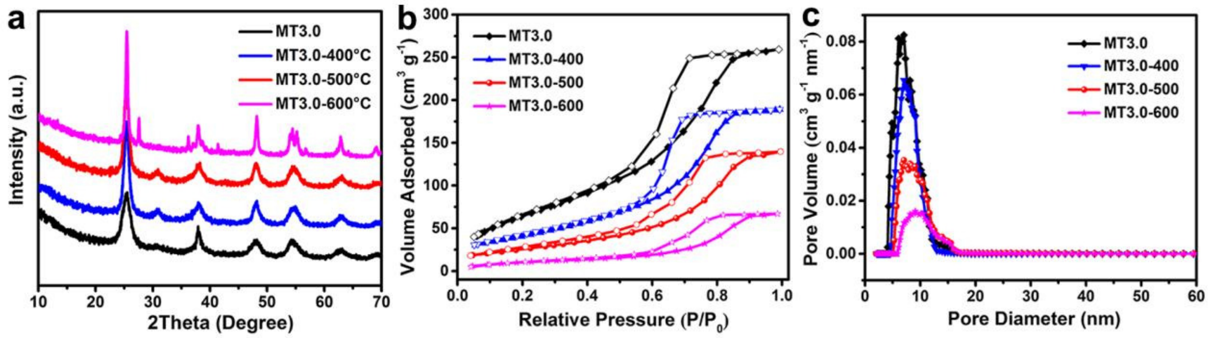

Figure S17. XRD patterns (a), nitrogen sorption isotherms (b), and pore size distributions (c) of MT3.0 calcined at different temperature in air.

Table S7 Structural properties of MT3.0 calcined at different temperature in air.

Temperature (T °C)	S_{BET} [a] (m^2 g^{-1})	Pore size (nm)	V_T [b] (cm^3 g^{-1})
400	152	7.0	0.293
500	101	7.0	0.216
600	40	9.1	0.103

Note: [a] BET surface area and [b] total pore volume.

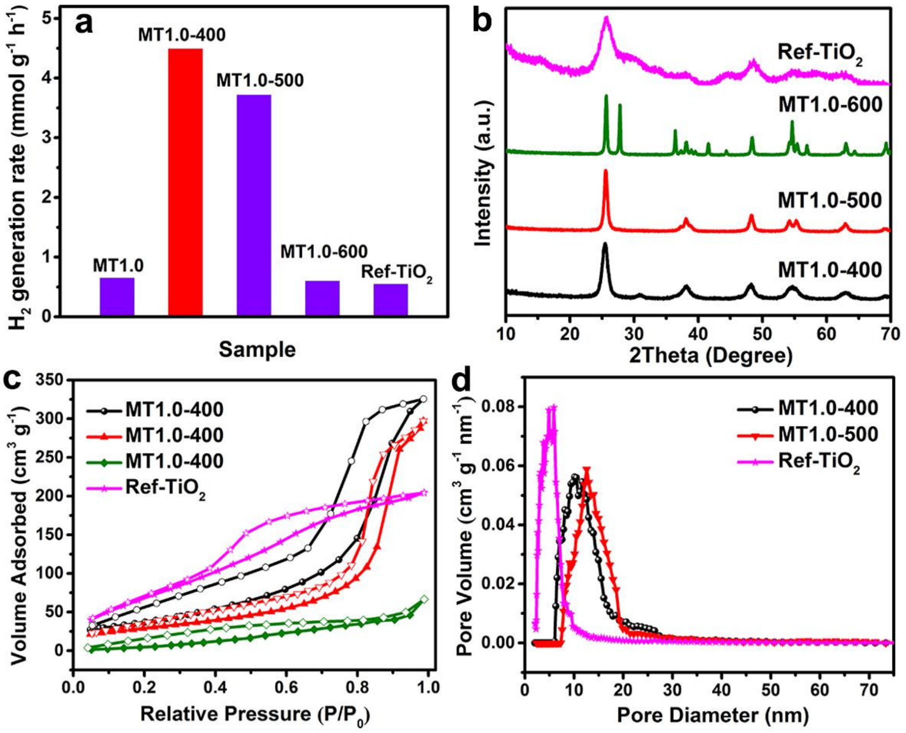

Figure S18. (a) Comparisons of average photocatalytic H_2 generation rates, XRD patterns (b), nitrogen sorption isotherms (c), and pore size distributions (d) of MT1.0 calcined at different temperature and Ref-TiO_2 in air.

Table S8 Structural properties of MT1.0 calcined at different temperature and Ref-TiO_2 in air.

Temperature (T °C)	S_{BET} [a] (m^2 g^{-1})	Pore size (nm)	V_T [b] (cm^3 g^{-1})
400	139	10.1	0.504
500	107	12.6	0.461
600	40	-	-
Ref-TiO_2	210	5.9	0.362

Note: [a] BET surface area and [b] total pore volume.

We have retested the photocatalytic performance of MT1.0 and MT1.0-T. Figure S18a showed the temperature-dependent photocatalytic H_2 generation rates using obtained MT1.0 sample as photocatalysts. The MT1.0 exhibited low photocatalytic activity due to its amorphous nature. When the calcination temperature was 400 °C, the MT1.0-400 exhibited high crystallinity, similar to MT3.0. With increasing the calcination temperature from 400 to 600 °C, the crystallinity of MT1.0 gradually

improved while the corresponding S_{BET} decreased from 139 m^2 g^{-1} to 39 m^2 g^{-1} (Figure S18b-d and Table S8). The H_2 evolution rates increased with increasing calcination temperature and reached a maximum at 400 °C, then decreased with further increasing the calcination temperature. The maximum H_2 evolution rate of 4.49 mmol h^{-1} g^{-1} for MT1.0-400 was similar to that of MT3.0-500 and higher than those of MT1.0 and Ref-TiO_2 prepared according to the literature,[S8] which may be attributed to high crystallinity of MT1.0-400. Furthermore, the photocatalytic activity of MT1.0-400 was higher than that of MT1.0-500 and MT1.0-600, which was attributed to large surface area of MT1.0-400.

Supplementary references

[S1] J. Zhang, Y. Deng, D. Gu, S. Wang, L. She, R. Che, Z. Wang, B. Tu, S. Xie, D. Zhao, Adv. Energy Mater. **2011**, 1, 241.

[S2] W. Zhou, W. Li, J. Wang, Y. Qu, Y. Yang, Y. Xie, K. Zhang, L. Wang, H. Fu, D. Zhao, J. Am. Chem. Soc. **2014**, 136, 9280.

[S3] B. Liu, L. Liu, X. Lang, H. Wang, X. Lou, E. S. Aydil, Energy Environ. Sci. **2014**, 7, 2592.

[S4] H. Zhu, Y. Shang, Y. Jing, Y. Liu, Y. Liu, A. El-Toni, F. Zhang, D. Zhao, ACS Appl. Mater. Interfaces **2016**, 8, 25586.

[S5] Y. Liu, R. Che, G. Chen, J. Fan, Z. Sun, Z. Wu, M. Wang, B. Li, J. Wei, Y. Wei, G. Wang, G. Guan, A. A. Elzatahry, A. A. Bagabas, A. M. Al-Enizi, Y. Deng, H. Peng, D. Zhao, Sci. Adv. **2015**, 1:e1500166.

[S6] Y. Liu, K. Lan, S. Li, Y. Liu, B. Kong, G. Wang, P. Zhang, R. Wang, H. He, Y. Ling, A. M. Al-Enizi, A. A. Elzatahry, Y. Cao, G. Chen, D. Zhao, J. Am. Chem. Soc. **2017**, 139, 517.

[S7] K. Lan, R. Wang, W. Zhang, Z. Zhao, A. Elzatahry, X. Zhang, Y. Liu, D. Al-Dhayan, Y. Xia, D. Zhao, Chem **2018**, 4, 1.

[S8] J. Fan, S. W. Boettcher, G. D. Stucky, Chem. Mater. **2006**, 18, 26.
